# Self‐determination, restrictive eating, and psychological needs: Challenges for young athletes

**DOI:** 10.1002/brb3.2761

**Published:** 2022-10-06

**Authors:** Camille Clermont, Linda Paquette, Daniel Lalande, Jacinthe Dion

**Affiliations:** ^1^ Intersectoral center for sustainable health, Department of Health Sciences Université du Québec à Chicoutimi Saguenay Québec Canada; ^2^ Centre de recherche interdisciplinaire sur les problèmes conjugaux et les agressions sexuelles (CRIPCAS) [Interdisciplinary Research Center on Intimate Relationship Problems and Sexual Abuse] Universitè de Montrèal 90 av. Vincent d'Indy Montréal, Quèbec H2V 2S9 Canada

**Keywords:** athletes, psychological needs, restrictive eating, self‐determination

## Abstract

**Objective:**

The purpose of this study was to examine the mediating role of self‐determination for sport in the relationship between psychological needs in a sport context, and restrictive eating behaviors among adolescent athletes, while controlling for confounding variables.

**Method:**

Self‐report online surveys were completed by 983 adolescent athletes (41.3% identified as girls, *M* = 14.63 years, *SD* = 0.765). Structural equation modeling was conducted to investigate the hypothesized associations between basic psychological need satisfaction and frustration in sport, self‐determination for sport, and restrictive eating behaviors, controlling for the level of competition and the type of sport practiced. Gender differences between the associations were assessed using moderation analysis.

**Results:**

Girls reported significantly more restrictive eating behaviors (*M*_girls_ = 0.85; *SD* = 1.39 vs. *M*_boys_ = 0.62; *SD* = 1.31). Both associations between basic psychological need frustration and satisfaction and restrictive eating behaviors were mediated by self‐determination for sport (*β* = 0.054, 95% bootstrap IC = 0.027–0.089; *β* = −0.045, 95% bootstrap IC = −0.079 to −0.021). Further, gender moderated an association so that frustration of basic psychological in sport more strongly predicted restrictive eating behaviors in girls (*β* = 0.256; *p* = 0.008), compared with boys.

**Discussion:**

Our study reveals considerable gender differences in the mechanism underlying the adoption of restrictive eating in adolescent athletes. The research also fills a gap in the literature by supporting the assumptions of the Hierarchical Model of Intrinsic and Extrinsic Motivation in eating behaviors, specifically in the sport context.

## Graphical Abstract

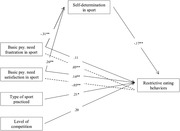

## 1 INTRODUCTION

Disordered eating, conceptualized as a continuum of maladaptive eating and weight control behaviors and attitudes (Bonci et al., 2008), is relatively common in adolescents (Neumark‐Sztainer et al., 2007). Disordered eating includes some of the same behaviors commonly associated with eating disorders, but they are not as frequent or severe (Gottlieb, 2014). Nevertheless, negative health outcomes are associated with disordered eating in adolescence (Herpetz‐Dahlmann et al., 2008; Jonhson et al., 2002; Santos, 2007), and behavioral characteristics of restrictive eating (i.e., limiting the consumption of calories and/or nutrients in a manner deleterious to long‐term health) are significantly related to risk for onset of recurrent binge eating and bulimia pathology (Stice et al., 2008).

Susceptibility of athletes to these problems is a serious concern: athletes experience higher rates of disordered eating than non‐athletes (Petrie & Greenleaf, 2011). An elite level of competition and being involved in weight‐sensitive sports appear to increase the risk (Krentz & Warschburger, 2011; Sundgot‐Borgen & Torstveit, 2010). Although it is important to examine components of sport participation in relation to eating behaviors, we believe it is equally important to consider the self‐determination processes and psychological needs involved in the practice of sports, an issue that can be addressed using self‐determination theory (SDT; Ryan & Deci, 2000).

SDT is an empirically derived approach to human motivation that proposes three basic psychological needs as predictors of optimal physiological, psychological, and social functioning: competence, autonomy, and relatedness (Ryan & Deci, 2000). Psychological needs are considered the psychological nutriments for growth and integration (Ryan & Deci, 2000). When people's needs are satisfied (i.e., when individuals feel competent, autonomous, and related to others), they thrive. Specifically, satisfaction of needs has been associated with greater well‐being (e.g., life satisfaction, vitality), reduced psychosocial concerns (e.g., depression, anxiety) and better overall health (Ryan & Deci, 2008; Vasteenkiste et al., 2010). Moreover, individuals develop greater self‐determination when their psychological needs are met (Verstuyf et al., 2012). Specifically, individuals with high self‐determination tend to act based on their interests, values, and goals, while low self‐determination reflects the extent to which the person focuses on the pressures and expectations from their social environment (Vansteenkiste et al., 2010). Lastly, basic psychological need frustration may be experienced when the individual feels oppressed, inadequate, or rejected. Defensive or self‐protective psychological adaptations may develop when psychological needs are frustrated (Ryan & Deci, 2000), which can have severe costs for health and well‐being (Adie et al., 2008). Such psychological adaptations include the development of need substitutes, that is, goals that people work toward to provide collateral satisfaction (Verstuyf et al., 2012), and compensatory behavioral patterns (Ryan, et al., 2006) that may involve alcohol abuse (Knee & Neighbors, 2002), smoking (Heatherton & Baumeister, 1991), binge eating or self‐control reinforcement, such as restrictive eating (Verstuyf et al., 2012).

Incorporating the fundamental tenets of SDT, Vallerand (2007) has proposed a Hierarchical Model of Intrinsic and Extrinsic Motivation (HMIEM). This model proposes that self‐determination processes mediate the relation between psychological needs, and affective, cognitive, and behavioral outputs. Beyond theoretical conjecture, several studies on eating behaviors have examined this sequence (Astani, 2015; Begin et al., 2018; Campbell et al., 2018; Froreich et al., 2017; Han & Lee, 2017; Paulisova et al., 2021; Schüler & Kuster, 2011; Thogersen‐Ntoumani et al., 2010; Verstuyf et al., 2013). For instance, Begin and her colleagues (2018) found that the satisfaction of psychological needs was positively associated with general self‐determination, which in turn was negatively associated with disordered eating behaviors in women from a population of university students and employees. Nevertheless, little is known about the role of the psychological needs and self‐determination in the sport context in the adoption of eating behaviors. To our knowledge, only two studies have addressed this issue, by measuring variables of interest in the sport context, and found that lower levels of self‐determination for sport (e.g., practicing primarily to improve one's shape, weight, and physical attractiveness) were associated with a greater risk of developing eating disorders in samples comprising mainly adult athletes (Chaba et al., 2021; Homan et al., 2019). However, to date, no research has investigated the relationships between psychological needs and self‐determination in sport, and eating behaviors in adolescent athletes, despite adolescence being a crucial development stage where changes in body image can influence eating behaviors (Baker et al., 2012). Further, gender differences regarding these associations have not been addressed.

Therefore, the current study aimed to test the assumptions of the HMIEM in the sport context and in relation to disordered eating, specifically restrictive eating. Based on the assumptions of the HMIEM, we expected that satisfaction of psychological needs in sport would be related positively to self‐determination for sport, whereas a negative association was anticipated with frustration of psychological needs in sport. It was also hypothesized that self‐determination for sport would be negatively associated with restrictive eating behaviors and that self‐determination for sport would mediate the relationship between psychological needs and restrictive eating. Given that the level of competition and the type of sport show associations with disordered eating in athletes (Krentz & Warschburger, 2011; Sundgot‐Borgen & Torstveit, 2010), those variables were included as control variables in our model. Lastly, we expected gender to moderate associations because eating behaviors may present themselves differently among athletes depending on gender (Baum, 2012). For instance, eating behaviors in boys may be related to their desire to be more muscular rather than for being thin (Selvi & Bozo, 2020).

## 2 METHOD

### 2.1 Participants

The study sample was drawn from an ongoing Canadian longitudinal study of sport participation and resilience that began in 2019. Of the 1802 participants in the overall study, only those who reported practicing organized sport and who completed the questionnaires about basic psychological needs, self‐determination for sport, and restrictive eating behaviors were included in this study, resulting in a final sample size of 983. Participants were aged between 14 and 18 years (*M*_age_ = 14.63 years, *SD* = 0.765) and engaged in sport since the age of about 8 (*M* = 7.89, *SD* = 3.641). Athlete's sociodemographic characteristics are presented in Table 1.

**TABLE 1:** Sociodemographic characteristics of participants

Characteristic	*n*	%
Sex assigned at birth (*n* = 983)		
Girls	403	41.0
Boys	580	59.0
Gender and sexual identity (*n* = 981)		
Girls	405	41.3
Boys	571	58.2
Non‐binary person	5	0.5
Cultural identity (*n* = 980)		
××x (blinded for review)	673	68.7
Canadian	89	9.1
Other cultural identities	218	22.2
Type of sport (*n* = 923)		
Endurance	112	12.1
Power/technical	44	4.8
Ball game	372	40.3
Weight dependent	42	4.6
Technical	2	0.2
Aesthetic	130	14.1
High mass	221	23.9
Competition level (*n* = 977)		
No competition	88	9.0
Local, regional, and inter‐regional	409	41.8
Provincial	363	37.2
National and international	117	12.0
Hours of practice (*n* = 977)		
10 or less	565	57.8
Between 11 and 15	196	20.1
16 or more	216	22.1

### 2.2 Procedure

Recruitment was conducted in two distinct phases—recruitment of school and participant recruitment within selected schools. Schools were contacted to participate in the study by the research coordinator, after a detailed explanation of the research project. To ensure sample diversity, the study recruited adolescents from Canadian schools in urban, semi‐urban, and rural areas, and from different socioeconomic backgrounds. Seven schools were invited to participate in the study and six of them agreed to participate. Data collection took place between October and December 2019. Participants had to meet the following inclusion criteria: (1) at least 14 years old, (2) in Grade 9 or 10, and (3) attending French or English high school. Therefore, a convenience sample of 1900 students was selected, and a participation rate of 97.7% was reached (only 43 students refused to participate). Out of the 1900 adolescents who participated, 11 participants were excluded because they were ineligible (less than 14 years old); 40 participants because they failed all of the three attention‐testing questions; and 4 participants because their answers appeared invalid (e.g., giving inconsistent answers to several questions), resulting in a final sample of 1802 adolescents. The research procedure was approved by the (blinded for review) Institutional Review Board and was conducted in accordance with the Declaration of Helsinki. Participants completed a self‐reported anonymous survey (Qualtrics Research Suite) in their classrooms on tablets provided by the research assistants. Prior to enrollment, participants received detailed information about the study and provided their informed consent.

### 2.3 Measures

#### 2.3.1 Sociodemographic

Questions about participants’ sport activities were used to obtain information about their current sport participation including the sport practiced, the weekly number of hours of sports and the level at which they were participating. Using Rosenvinge et al.’s (2018) work, we classified practiced sports into seven categories, namely, endurance, power/technical, ball game, weight dependent, technical, aesthetic, and high mass. A dichotomous variable was computed to distinguish weight‐sensitive sports from less weight‐sensitive sports. Sports where physical appearance is a fundamental component of judging criteria (i.e., aesthetic sports), sports where athletes’ weight determines the category in which they will compete (i.e., weight‐dependent sports) and sports where low body weight may enhance performance (i.e., technical and endurance sports) were considered weight‐sensitive sports in the present study (Bar et al., 2016).

General sociodemographic information was collected (age, sex assigned at birth, cultural identity, and gender). Gender was assessed using the question: “What is the gender or sex that is yours or that you feel to be yours (your gender identity)?”

#### 2.3.2 Basic psychological need satisfaction and frustration

The athletes’ satisfaction and frustration of psychological needs in their sport was assessed using the Basic Psychological Needs Satisfaction and Frustration Scale (BPNSFS; Chen et al., 2015), which has been used successfully in previous research with adolescents (Campbell et al., 2018). A 16‐item ××x shortened version (blinded for review) was used for the present study, of which nine items tapped into need satisfaction and seven into need frustration. Based on previous practices (Behzadnia et al, 2018; Haerens et al., 2015; Rodrigues et al., 2018; Tilga et al., 2018), this general need satisfaction and frustration scale was adjusted by adding the stem “When answering the following items, think about how you feel when you practice your sport. In the context of my sport…” and by slightly rewording one item to better reflect the specific context of sport (“I feel excluded from the group I want to belong to” was changed into “I feel excluded from my team”). Participants rated the items on a five‐point Likert scale (from 1 = ‘‘not true at all,’’ to 5 = ‘‘completely true’’), with higher scores reflecting higher levels of need satisfaction and frustration. The subscales of the BPNSFS demonstrated good reliability with Cronbach alphas of 0.834 and 0.762, respectively, in the present sample.

#### 2.3.3 Self‐determination for sport

Participants’ self‐determination for their sport was assessed using an adapted and shortened ××x version (blinded for review; i.e., 15 items out of 19) of the Behavioral Regulation in Exercise Questionnaire‐2 (BREQ‐2) (Markland & Tobin, 2004). Each type of regulation (external, introjected, identified, and intrinsic) was assessed with three items. The amotivational factor was not considered in the present study because the participants were athletes and amotivation is the state in which an individual lacks the intention to act (Deci & Ryan, 2000). Based on previous research (Scoffier‐Mériaux et al., 2021), this exercise regulation scale was adjusted by adding the stem “Evaluate your motivation to practice sport” and by slightly rewording some items to better reflect the specific context of sport. To illustrate, the item “I feel guilty if I don't exercise” was changed into “I feel guilty if I don't practice sport.” Each item was measured on a five‐point Likert scale (from 0 = ‘‘not true for me,’’ to 4 = ‘‘very true to me’’). A self‐determination index was also calculated by weighting external regulation “−2″, introjected regulation ”−1″, identified regulation “+1″, and intrinsic motivation ”+2″, with higher scores reflecting higher self‐determination for sport (Grolnick & Ryan, 1987). In our sample, this scale demonstrated good reliability with a Cronbach's alpha of .727.

#### 2.3.4 Restrictive eating behaviors

Restrictive eating behaviors were measured using the dietary restriction subscale of the validated ××x short form (blinded for review) of the Eating Disorder Examination Questionnaire (EDE‐Q) (Fairburn & Beglin, 1994). Participants were asked to indicate how often each of three events occurred on a seven‐point Likert scale, ranging from 0 (no days) to 6 (every day) during the last 28 days. A sample item included: “For how many of the past 28 days have you intentionally tried to limit the amount of food you eat to influence your shape or weight (whether successful or not)?”. The score was determined by averaging the score of the three items, with higher scores reflecting greater restrictive eating behaviors. In the current study, the scale showed good reliability with a Cronbach's alpha of .862.

### 2.4 Statistical analyses

Descriptive statistics, independent samples *t*‐tests, correlations, and Cronbach's alphas were computed using SPSS 26. The assumptions of multivariate analyses were verified before proceeding to structural equation modeling (SEM). Because all assumptions, apart from normality, were met, we used the maximum‐likelihood (MLR) estimator which can provide fit indices and standard errors that are robust with non‐normal distributions. Missing data for examined variable were minimal and were handled using the full information likelihood estimation (FIML). Lastly, the sample size required for the study was determined based on simulation research (Fritz & MacKinnon, 2007), which indicated that a minimum sample size of 462 participants was required to detect a mediation effect when both coefficients defining the interaction are small (e.g., *β* ≤ 0.14).

Mplus 8 was used to conduct SEM (Muthén & Muthén, 1998–2017). We examined the mediating role of self‐determination for sport in the associations between psychological needs satisfaction and frustration in sport and the frequency of restrictive eating, controlling for confounding variables. First, the model was examined with the entire sample, without the control variables (Model 1). Then, the control variables (i.e., competitive level and type of sport) were added to the model (Model 2). Finally, the moderating role of gender was tested (Model 3) and the indirect effects were added to the model (Model 4). Indirect effects were tested by bias‐corrected bootstrapping (10,000 bootstrap replication samples) using 95% confidence intervals (CI; Ferguson, 2016; Preacher & Hayes, 2008; Schellenberg et al., 2016). Commonly used goodness‐of‐fit indices were examined to assess acceptability of models. Specifically, the Comparative Fit Index (CFI; ≥0.90 for acceptable; ≥0.95 for excellent), the Tucker–Lewis index (TLI; ≥0.90 for acceptable; ≥0.95 for excellent), and root‐mean‐square error of approximation (RMSEA; ≤0.08 for acceptable; ≤0.06 for excellent) with 90% CI were used (Bentler, 1990; Browne & Cudeck, 1993; Marsh et al., 2005; Schermelleh‐Engel et al., 2003).

## 3 RESULTS

Table 2 presents the associations between psychological needs satisfaction and frustration in sport, self‐determination for sport, restrictive eating behaviors, type of sport practiced, and level of competition.

**TABLE 2:** Pearson's correlation coefficients for study variables

Variable	Range	*M* (*SD*)	1	2	3	4	5
1. Basic needs satisfaction in sport	0–4	3.19 (0.63)					
2. Basic needs frustration in sport	0–4	1.18 (0.73)	−0.33**				
3. Self‐determination for sport	−12–12	8.18 (2.66)	0.35**	−0.39**			
4. Restrictive eating behaviors	0–6	0.72 (1.35)	−0.03	0.23**	−0.20**		
5. Type of sport practiced	0–1	0.32 (0.46)	0.01	−0.03	−0.07*	0.07*	
6. Level of competition	0–1	0.12 (0.46)	0.08*	0.03	0.05	0.12**	0.14**

*Note*. *M*, mean; *SD*, standard deviation. The type of sport practiced and the level of competition were dummy coded (type of sport practiced: 0 = less weight‐sensitive sports, 1 = weight‐sensitive sports; level of competition: 0 = no competition, local, regional interregional, and provincial level; 1 = national and international level).

**p ≤ 001; *p ≤ 05.

Comparisons of scores by gender are presented in Table 3. We observed significant differences for all variables, except for self‐determination for sport and competition level. Adolescent girls reported significantly more restrictive eating behaviors and higher levels of psychological needs frustration in sport, alongside lower levels of psychological needs satisfaction in sport than boys. However, the magnitude of the differences found was small. Furthermore, adolescent girls engaged more in weight‐sensitive sports compared with adolescent boys and the magnitude of the difference found was large.

**TABLE 3:** Means, standard deviations and standard mean differences for study variables by gender

		Boys		Girls	
Variable	*d*	*M*	*SD*	*M*	*SD*
Basic needs satisfaction in sport	0.222	3.25	0.63	3.11	0.61
Basic needs frustration in sport	−0.256	1.13	0.74	1.24	0.72
Restrictive eating behaviors	−0.165	0.62	1.31	0.85	1.39
Type of sport practiced	−0.725	0.18	0.39	0.50	0.50

*Note*. *M*, mean; *SD*, standard deviation; *d*, standard mean differences. The differences between the variables were significant (*p* ≤ .05). Type of sport practiced was dummy coded (0 = less weight‐sensitive sports, 1 = weight‐sensitive sports).

**FIGURE 1:**
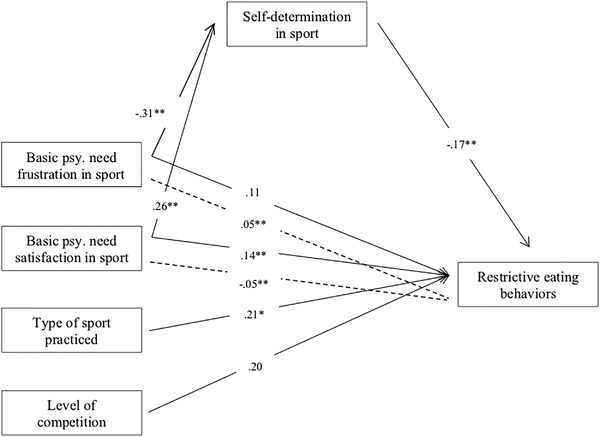
Associations between basic psychological need frustration and satisfaction in sport, self‐determination in sport and restrictive eating behaviors, controlling for the type of sport practiced, and the level of competition (Model 4). Note. One headed arrow represents standardized regression weights. Dashed arrows indicated indirect pathways. For the sake of clarity, moderating gender effect are not depicted. Type of sport practiced and level of competition were dummy coded (type of sport practiced: 0 = less weight‐sensitive sports, 1 = weight‐sensitive sports; level of competition: 0 = no competition, local, regional interregional and provincial level; 1 = national and international level). Paths with corresponding significant standardized coefficient are represented with asterisks. *p ≤ .05 **p ≤ .01

All estimated SEMs demonstrated an acceptable to excellent fit (Table 4). The results of Model 4 are presented in Figure 1. Basic psychological need frustration (*β* = −0.312; *p* ≤ .001) and satisfaction (*β* = 0.260; *p* ≤ .001) in sport predicted self‐determination for sport. Self‐determination for sport, in turn, negatively predicted restrictive eating behaviors (*β* = −0.174; *p* ≤ .001). Direct (*β* = 0.143; *p* = .003) and indirect effects (*β* = −0.045, 95% bootstrap IC = −0.079 to 0.021) were found between basic psychological need satisfaction in sport and restrictive eating behaviors. No significant direct effect was found between psychological need frustration in sport and restrictive eating behaviors (*β* = 0.109; *p* = .080), but the indirect effect pathway was significant (*β* = 0.054, 95% bootstrap IC = 0.027–0.089). We also added control variables to our model related to sport, such as level of competition and the type of sport practiced. Being an elite athlete (i.e., competing at a national or international level) did not predict restrictive eating behaviors in our sample (*β* = 0.198; *p* = .087). However, participants who practiced weight‐sensitive sports showed a higher frequency of restrictive eating behaviors (*β* = 0.205; *p* = .014).

**TABLE 4:** Comparison of the associations between basic needs satisfaction/frustration in sport, self‐determination for sport, and restrictive eating behaviors across boys and girls, controlling for type of sport and competitive level

Models	*χ* ^2 (df)^	CFI	TLI	RMSEA	90% C.I.
Model 1: Model without control variables	0 (0)	1.000	1.000	0.000	0.000–0.000
Model 2 : Model with control variables	23.29 (6)	0.916	0.874	0.055	0.033–0.079
Model 3: Model with control variables, adding the moderating role of gender	9.852 (5)	0.980	0.941	0.033	0.000–0.063
Model 4: Same as Model 3, adding the indirect effects	9.852 (5)	0.980	0.941	0.033	0.000–0.063

Abbreviations: *χ* ^2^, Chi‐square; df, degrees of freedom; CFI, Comparative Fit Index; TLI, Tucker–Lewis Index; RMSEA, root‐mean‐square error of approximation, 90% CI = 90% confidence interval of the RMSEA.

**FIGURE 2:**
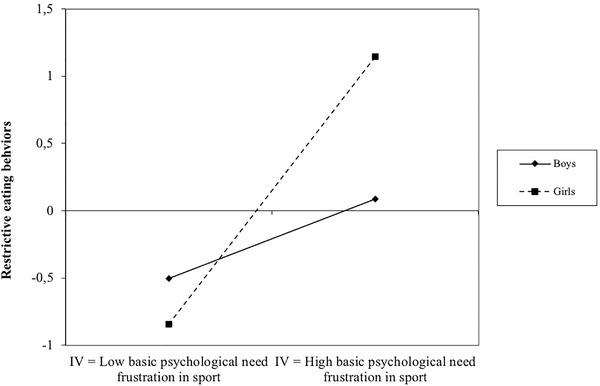
Moderating role of gender in the relationship between frustration of psychological needs in sport and restrictive eating behaviors. *Note*. Restrictive eating behaviors and basic psychological need frustration in sports have been standardized

Moreover, the study assessed the moderating role of gender on the relationship between basic psychological needs in sport and restrictive eating behaviors. The model explained 8.9 % of the variance of restrictive eating behaviors without inclusion of the moderation effect. With its inclusion, the variance explained increased to 12.1%. Further, significance of moderating effects was analyzed. The results revealed that gender moderated the association between psychological need frustration (*β* = 0.256; *p* = .008) in sport and restrictive eating behaviors. As shown in Figure 2, frustration of basic psychological needs in sport more strongly predicted restrictive eating behaviors in girls, compared with boys. No moderation effect of gender was found in the association between satisfaction of psychological need in sport and restrictive eating behaviors (*β* = −0.062; *p* = .449).

## 4 DISCUSSION

The present study aimed to improve our understanding of restrictive eating behaviors in athletes by testing the associations between satisfaction and frustration of psychological needs in sport, self‐determination for sport, and restrictive eating behaviors. The level of competition and the type of sport practiced were controlled for. Based on the HMIEM, a model was elaborated and the moderating role of gender in the associations was tested among adolescent athletes.

### 4.1 Evidences for the HMIEM

First, satisfaction of psychological need in sport was positively and significantly related to restrictive eating in our sample. While this result is inconsistent with our assumption, previous research has reached a similar conclusion that low levels of basic psychological need satisfaction did not significantly predicted ill health in athletes (Gagné, 2003; Quested & Duda, 2010). Speculatively, restrictive eating may stem from perceived psychological need satisfaction in sport because athletes may diet to improve athletic performance (Greenberg & Schoen, 2008). However, the indirect association between the satisfaction of psychological needs and restrictive eating via self‐determination for sport was negative, which is in line with the assumptions of the HMIEM. Altogether, these results suggest that self‐determination for sport has an inconsistent mediation effect. Further research is needed to better understand the mechanisms underlying these relations.

As hypothesized, the results revealed a full mediation effect of self‐determination for sport in the association between frustration of psychological need in sport and restrictive eating behaviors. This finding is in line with prior studies in adults that found that individuals tend to develop need substitutes when their psychological needs are frustrated. Specifically, need frustration have been positively related to a drive for muscularity (Selvi & Bozo, 2020), muscle checking, and exercise dependency (Edwards et al., 2016) and a recent study showed that such goals are associated with poor self‐determination for sport (Chaba et al., 2019). Therefore, our result provide evidence for the assumptions of the HMIEM in the sport context and in relation to eating behaviors in adolescents, adding to the existing literature that corroborates these associations in several life domains (Gagné & Deci, 2005; Grolnick et al., 1997; Hager & Chatzisarantis, 2007; Sarrazin et al., 2006; Williams et al., 2002).

The present study also revealed a significant negative association between self‐determination for sport and restrictive eating behaviors in adolescent athletes. This result supports the findings of previous studies conducted mainly in adults. For example, lower self‐determination for sport has been associated with a greater risk of developing eating disorders in female and male athletes (Chabba et al., 2021; Homan et al., 2019). Additionally, Mond and his colleagues (2009) found that exercising primarily to improve one's shape, reduce weight, and enhance physical attractiveness, which represent low self‐determination, distinguished between women with eating disorders and healthy women.

Further, being an elite athlete (i.e., competing at a national or international level) did not predict restrictive eating behaviors in our sample. While this result is inconsistent with our assumption, previous research has reached a similar conclusion in a sample of adolescent athletes (Karrer et al., 2020). Lastly, participants who practiced weight‐sensitive sports showed higher frequencies of restrictive eating behaviors in our study, consistent with previous research (Krentz & Warschburger, 2011).

### 4.2 Gender differences

We found that psychological needs frustration in sport more strongly predicts restrictive eating behaviors in girls, compared with boys. Speculatively, restrictive eating may stem more strongly from psychological need frustration in girls, because of their desire to be thin (Selvi & Bozo, 2019). In contrast, boys’ body image‐related problems are typically associated with desire for a more muscular body (Pope et al., 2000) and restrictive eating may not be the right way to achieve this goal. Further studies are needed to better understand psychological adaptations used among boys to cope with the frustration of their psychological needs in sport. In contrast, no significant moderation effect of gender was found in the association between satisfaction of psychological need and restrictive eating behaviors. More studies are needed to corroborate this finding since gender differences in this association has not been addressed by previous research to our knowledge.

### 4.3 Limitations

Some limitations need to be highlighted. First, the associations found between variables do not demonstrate any causal link because the cross‐sectional design does not permit any inference about causality. Besides, although this study used a large and diverse sample, it was not representative of all adolescents in the country, limiting the generalizability of the findings. In addition, problematic eating behaviors in this study refer to restrictive eating, measured by the dietary restriction subscale (Machado et al., 2020) of the EDE‐Q (Fairburn & Beglin, 1994). Therefore, the results cannot be extrapolated to other eating behavior problems, such as compulsive eating or binge eating. Moreover, since the instruments used to measure basic psychological needs in sport and self‐determination for sport were adapted slightly for the sport context by the research team, further studies should replicate these findings using other measures of these constructs. Lastly, constructs were tested with self‐reported measures, and therefore, the results may have been influenced by social desirability.

### 4.4 Clinical and theoretical implications

Despite these limitations, our study has important implications. The results suggest that a global conceptual model based on the HMIEM may help to improve our understanding of restrictive eating behaviors and of gender differences among those affected. More precisely, our results have revealed that there is a considerable difference among gender in the mechanism underlying the adoption of restrictive eating in adolescent athletes. The study also fills a gap in the literature by supporting the assumptions of the HMIEM in the sport context and in relation to eating behaviors. Therefore, professionals who guide young athletes in the regulation of their eating behaviors may benefit from more thorough consideration of self‐determination and psychological needs specifically in the sport context. Moreover, both frustration and satisfaction of psychological needs in sport should be investigated because restrictive eating was associate with greater satisfaction of psychological needs in the present study. Thus, frustration of psychological needs in sport may be a better indicator of restrictive eating behaviors, especially in girls.

## CONFLICT OF INTEREST

The authors declare no conflict of interest.

### PEER REVIEW

The peer review history for this article is available at https://publons.com/publon/10.1002/brb3.2761

## Data Availability

The data that support the findings of this study are available from the first and last authors. Although restrictions apply to the availability of these data, which were used under license for the current study and are therefore not publicly available, they are available upon request.
